# Both Knotless and Traditional Knot‐Tying Suture Anchors Result in Favorable Clinical Outcomes in the Treatment of Acetabular Labral Tears: A Systematic Review

**DOI:** 10.1002/ars2.70092

**Published:** 2026-09-28

**Authors:** David Hou, Laura Ndjonko, Samuel R. Johnson, Patrick England, Ujash Sheth, Vehniah K. Tjong

**Affiliations:** ^1^ Department of Orthopaedic Surgery Northwestern University Feinberg School of Medicine Chicago Illinois U.S.A.; ^2^ Department of Orthopaedic Surgery New York University Grossman School of Medicine New York City New York U.S.A.; ^3^ Sunnybrook Orthopaedic Upper Limb (SOUL), Sunnybrook Health Sciences Centre University of Toronto Ontario Canada

## Abstract

**Purpose:**

To compare clinical outcomes and complication profile of knotless versus knot‐tying suture anchors in arthroscopic hip labral repair.

**Methods:**

A systematic review was performed following Preferred Reporting Items for Systematic reviews and Meta‐Analysis guidelines. Data on suture anchor type, patient‐reported outcomes (including modified Harris Hip Score, Hip Outcome Score‐Activities of Daily Living, Hip Outcome Score‐Sport Specific Subscale, Visual Analog Scale, International Hip Outcome Tool‐12), complications, and concomitant procedures were extracted. Bias was assessed via Risk of Bias in Non‐randomized Studies and Revised Cochrane Risk‐of‐Bias Tool for Randomized Trials.

**Results:**

A total of 10 studies met all the inclusion criteria, including 2 studies that directly compared knotless with knot‐tying suture anchors. All studies had a minimum of 12‐month follow‐up. Both knotless and knot‐tying suture anchors showed significant improvement across all measured patient‐reported outcomes (mean modified Harris Hip Score preop‐postop: knotless vs knot‐tying =61.0‐84.8 vs 68.6‐85.9; mean Hip Outcome Score‐Activities of Daily Living preop‐postop: knotless vs knot‐tying = 52.3‐79.1 vs 51.4‐77.7; mean Hip Outcome Score‐Sport Specific Subscale preop‐postop: knotless vs knot‐tying = 44.9‐76.1 vs 49.3‐77.9; mean Visual Analog Scale preop‐postop: knotless vs knot‐tying = 5.6‐1.9 vs 5.6‐1.9; mean International Hip Outcome Tool‐12 preop‐postop: knotless vs knot‐tying = 35.4‐77.2 vs 37.4‐76.7). Across studies reporting patient‐level thresholds, high proportions of patients achieved clinically meaningful improvement, with minimal clinically important difference achievement ranging from approximately 74% to 100% and patient acceptable symptom state achievement ranging from 46% to 100% across outcome measures, with no consistent differences between knotless and knot‐tying anchor constructs. Complication, revision, and total hip arthroplasty conversion rates were low in both groups (revision rate range: knotless = 1.9%‐6.8%, knot‐tying = 0%‐12.8%; total hip arthroplasty conversion rate range: knotless = 2.6%‐5.7%, knot‐tying =0.0%‐4.7%). One study reported that postoperative synovitis was found in more patients in the knot‐tying group than in the knotless group (17 vs 5). In the included studies, there was risk of bias, including confounding bias (6/8 studies) and selection bias (7/8 studies).

**Conclusions:**

Knotless and knot‐tying suture anchors are both safe and effective options for hip labral repairs with anticipated improvement in patient‐reported outcomes regardless of the anchor type used. Across studies reporting patient‐level thresholds, a high proportion of patients achieved minimal clinically important difference and patient acceptable symptom state, supporting the clinical relevance of postoperative improvement in both constructs. There were no significant differences in revision rates or conversion to total hip arthroplasty and complications are low in both groups.

**Level of Evidence:**

Level IV, systematic review of Level II to IV studies.

## Graphical abstract

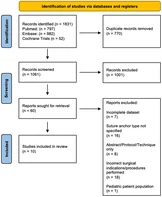

Acetabular labral tears are among the most common hip pathologies, comprising 22%‐55% of all diagnoses of hip pain in the clinical setting.^1^ Labral tears are often seen in the setting of femoral acetabular impingement (FAI) or hip dysplasia where underlying developmental abnormalities increase the risk of symptomatic labral tears.^2^, ^3^ In the nondegenerative hip, surgical management of a labral tear is often indicated in conjunction with the treatment of the underlying anatomical abnormality, such as an osteoplasty for cam or pincer morphology.^4^, ^5^

Several technical variations exist in the setting of arthroscopic labral repairs, including repair versus debridement, capsular management (the extent of capsulotomy and closure), traction techniques, concomitant procedures (i.e., microfractures, chondroplasty), and postoperative rehabilitation protocols.^6^, ^7^ Knotless suture anchors were first introduced in the early 2000s with the intent to reduce surgical time and material bulk in the joint.^8^ They have since been integrated into arthroscopic surgeries across a variety of different joints, including the hip, shoulder, knee, elbow, and ankle. Biomechanical studies comparing knotless versus knot‐tying suture anchors commonly cite disadvantages of knotless suture anchors having lower pull‐out strength and higher cost compared with traditional knot‐tying suture anchors.^9^, ^10^, ^11^ However, the clinical significance of these findings has not been fully elucidated. In the field of shoulder arthroscopy, knotless versus knot‐tying suture anchors in the repair of Bankart lesions and other labral tears have not shown significant difference in patient‐reported outcomes (PROs).^12^ Within hip arthroscopy, multiple studies examining the postoperative outcomes after labral repair have been published, but the difference between knotless versus knot‐tying suture anchors has not been comprehensively synthesized.

The purpose of this study was to compare clinical outcomes and complication profile of knotless versus knot‐tying suture anchors in arthroscopic hip labral repair. We hypothesized that PROs are similar to each anchor type, with both knotless and knot‐tying anchors possessing their own innate advantages and disadvantages.

## METHODS

This study was reported in accordance with the Preferred Reporting Items for Systematic reviews and Meta‐Analysis criteria. The study was Institutional Review Board exempt.

### Search Strategy

An electronic literature search was performed using PubMed, Embase, and Cochrane Trials from database inception to May 2025. The Boolean query involved a combination of the following medical subject headings and related synonyms: (repair*[tiab]) AND (&“Hip”[Mesh] OR Hip[title/abstract] OR hips[tiab] OR pelvis[tiab] OR acetabul*[tiab] OR coxa[tiab] OR coxas[tiab]) AND (Labral*[tiab] OR labrum*[tiab]). Additional related studies were identified by reviewing the reference lists of eligible articles.

### Eligibility Criteria

Studies were considered for inclusion if they were categorized as a randomized controlled trial, cohort study, case control study, or case series evaluating PROs after primary arthroscopic acetabular labral repair in adults (age >17 years) where FAI was the main surgical indication. This review excluded studies in hip dysplasia, revision surgery, or associated surgeries such as debridement without repair, reconstructions, or periacetabular osteotomies. All studies had at least 10 patients and included data on PROs and type of suture anchor used. Studies not in English or without full texts and/or dataset were also excluded.

### Study Selection

Relevant title and abstract screening were conducted independently by two of the coauthors (D.H., L.N.) to determine if the study met the inclusion criteria. Duplicate studies were removed and for any study whose inclusion eligibility was questionable, articles were promoted to the full‐text screening phase. If a conflict occurred at this phase, the senior author (V.K.T.) reviewed the study for inclusion.

### Data Extraction

This study collected data from articles comparing the PROs after arthroscopic acetabular labral repair. Extracted variables include patient demographics, type of suture anchor used, surgical indication and inclusion constraints, capsular management, concomitant procedures, postoperative rehabilitation protocol, all PROs, complications, and rates of revision surgery or conversion to total hip arthroplasty (THA). PROs included modified Harris Hip Score (mHHS), Hip Outcome Score‐Activities of Daily Living (HOS‐ADL), Hip Outcome Score‐Sport Specific Subscale (HOS‐SSS), Visual Analog Scale (VAS), International Hip Outcome Tool‐12, Nonarthritic Hip Score (NAHS), UCLA Activity Score, and Hip Disability and Osteoarthritic Outcome Score.

### Assessment of Risk of Bias in Eligible Studies

The risk of bias for each eligible randomized controlled trial was evaluated using the Revised Cochrane Risk‐of‐Bias Tool for Randomized Trials based on 5 domains: bias arising from the randomization process, deviations from intended interventions, missing outcome data, measurement of the outcome, and selection of the reported result.^13^ Each domain is rated as low risk, some concerns, or high risk of bias, leading to an overall risk of bias judgment for each study. Risk of bias for other studies was done with the Risk of Bias in Non‐randomized Studies, which evaluated domains of confounding, selection of participants, classification of interventions, deviations for intended interventions, missing data, measurement of outcomes, and selection of the reported result.^14^ These structured approaches ensure a comprehensive and standardized evaluation of bias in the included studies. Two independent raters assessed each paper and the senior author resolved all conflicts.

## RESULTS

### Literature Search

A Preferred Reporting Items for Systematic reviews and Meta‐Analysis flow diagram (Figure 1) depicts the results of the search strategy and criteria for inclusion. Our literature search generated 1831 potential studies for screening through 3 different databases. Duplicate screening removed 770 studies, and among the 60 studies that underwent full review, 50 were further excluded because of lack of specification of suture anchor type, incomplete datasets, incorrect surgery, and/or incorrect labral repair indications. After screening, 10 studies were deemed eligible and included for analysis.^15^, ^16^, ^17^, ^18^, ^19^, ^20^, ^21^, ^22^, ^23^, ^24^

**FIGURE 1:**
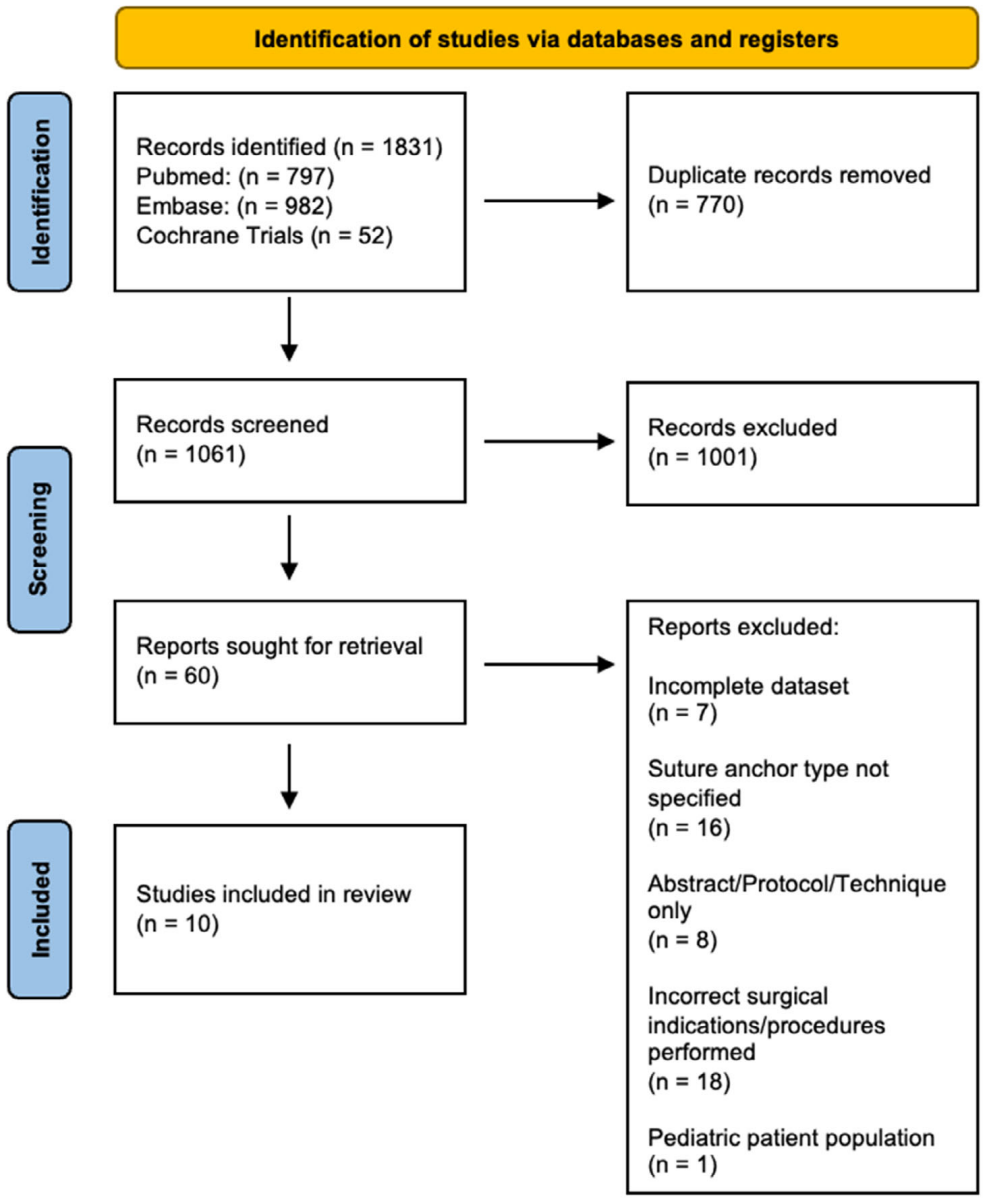
Consort diagram depicting search strategy and study selection process as set by the PRISMA guidelines. (PRISMA, Preferred Reporting Items for Systematic reviews and Meta‐Analysis.)

### Quality Assessment

Sources of bias were analyzed using the Cochrane risk assessment tool (Figure S1). Both randomized controlled trials, Rhee et al. and Economopoulos et al., were found to have low risk across all domains. Risk of Bias in Non‐randomized Studies tool showed moderate risk of confounding bias in 6 out of 8 nonrandomized studies because of a general lack of multivariate adjustment, whereas Su et al. and Maldonado et al. were graded as serious risk because of lack of adjustment for known baseline confounders. Selection bias was rated as moderate in 7 out of 8 nonrandomized studies because of study type, and Christoforetti et al. were graded as serious because it is a case series without any predefined comparisons. Su et al. and Maldonado et al. were graded as serious risk of missing data because of substantial attrition in follow‐up studies.

### Study and Patient Characteristics

Only 2 studies, Rhee et al. and Celiksoz et al., directly compared knotless and knot‐tying suture anchors with Rhee et al. being the only randomized control study. Most of the studies were conducted between 2010 to 2020 across multiple countries, including the United States, Republic of Korea, China, and Brazil (Table 1). Four studies characterized surgical outcomes using only knot‐tying suture anchors (Lim et al., Vassalo et al., Su et al., and Scanaliato et al.), and 4 studies used only knotless suture anchors (Economopoulos et al., Christoforetti et al., Maldonado et al., and Maldonado et al.) (Table 1).

**TABLE 1:** Study Information for Eligible Studies Identified After Literature Review. Ten Unique Studies Were Included in Our Review, Including 4 Studies With Only Knot‐Tying Suture Anchors, 4 Studies With Only Knotless Suture Anchors, and 2 Studies With Both Knotless and Knot‐Tying Suture Anchors

**Author (Year)**	**Level of Evidence**	**Surgery Dates**	**Average Follow‐Up, Months**	**Country**	**No. of Patients**	**Mean Age, Years**	**Sex, n**	**Suture Anchor Model**	**Suture Anchor Type**	**Mean No. of Anchors [SD]**	**Other Procedures, %**	**Capsular Repair**
Lim (2020)^18^	III, Cohort study	January 2010‐December 2016	49.5	Republic of Korea	22	39.8	15 F, 6 M	NA	Knot‐tying	2‐4 suture anchors	Femoroplasty (36.4%) Acetabuloplasty (9.1%) Other procedure (9.1%)	NR
Vassalo (2018)^24^	IV, Therapeutic case series	October 2010‐December 2013	43	Brazil	84	44.2	32 F, 52 M	2.7 mm anchors	Knot‐tying	1.6	Not reported	NR
Su (2024)^23^	III, Retrospective comparison study	January 2018‐December 2020	31.2	China	76	38.5	37 F, 39 M	Smith and Nephew 2.3 mm Osteoraptor suture anchors	Knot‐tying	NR	Femoroplasty (88.2%) Acetabuloplasty (52.6%) Chondroplasty (10.5%) Excision of acetabular bone cyst (6.6%) Ligamentum teres debridement (1.3%) Iliotibial band release (2.6%) Loose body removal (2.6%) Trochanteric bursectomy (1.3%)	100%
Scanaliato (2022)^22^	III, Cohort study	January 2015‐December 2015	60.12	USA	68	29.9	41 F, 27 M	Stryker Nanotack flex	Knot‐tying	NR	Femoroplasty (82.5%) Acetabuloplasty (23.5%) Chondroplasty (57.4%)	NR
Rhee (2016)^21^	II, Randomized control trial	September 2012‐May 2013	32.3	Republic of Korea	19	33.8	9 F, 10 M	Smith and Nephew Bioraptor suture anchors	Knot‐tying	2‐4 suture anchors	NR	NR
Celiksoz (2024)^15^	III, Cohort study	January 2017‐January 2021	25	Turkey	157	34	77 F, 80 M	Smith and Nephew 1.8‐mm knot‐tying Q‐FIX anchors	Knot‐tying	4 (median)	Cartilage debridement and microfracture (82.2%)	100%
Economopoulos (2020)^17^	II, Randomized control trial	July 2014‐March 2016	24	USA	150	36.9	61 F, 89 M	Arthrex knotless Suturetek	Knotless	NR	Femoroplasty (100%)	
Christoforetti (2021)^16^	III, Retrospective case series	July 2015‐October 2018	12	USA	245	30.1	137 F, 108 M	Arthrex PEEK 2.4‐mm knotless PushLock	Knotless	2.1 [0.67]	Femoroplasty (51%) Acetabuloplasty (38%) Synovectomy (57%) Ligamentum teres debridement (20%)	
Maldonado (2020)^19^	IV, Case series	February 2015‐January 2017	30.59	USA	309	36.2	211 F, 98 M	Arthrex 3.0‐mm PEEK Knotless Hip SutureTak suture anchors	Knotless	NR	Femoroplasty (100%) Acetabuloplasty (93.9%) Microfracture (8.7%) Ligamentum teres debridement (7.1%)	100%
Maldonado (2021)^20^	III, Case control study	January 2014‐June 2018	37.6	USA	106	48.6	50 F, 56 M	Arthrex 3.0‐mm PEEK Knotless SutureTak and/or Arthrex 1.8‐mm Knotless FiberTak anchors	Knotless	NR	Femoroplasty (100%) Acetabuloplasty (71.7%) Microfracture (16%)	61.30%
Rhee (2016)^21^	II, Randomized control trial	September 2012‐May 2013	31.8	Republic of Korea	18	34.6	13 F, 5 M	Smith and Nephew Bioraptor knotless suture anchors	Knotless	2‐4 suture anchors	NR	NR
Celiksoz (2024)^15^	III, Cohort study	January 2017‐January 2021	34	Turkey	256	35	122 F, 134 M	Arthrex 2.9‐mm knotless biocomposite anchors	Knotless	4 (median)	Cartilage debridement and microfracture (100%)	100%

F, female; M, male; NA, not applicable; NR, not reported; PEEK, polyetheretherketone; SD, standard deviation.

Additional procedures performed alongside labral repair varied across studies (Table 1). Femoroplasty was the most reported adjunct procedure, with frequencies ranging from 36.4% to 100% in studies that reported these data. Other procedures include acetabuloplasty, ligamentum teres debridement, and microfracture, although frequencies of these procedures also ranged widely among studies. Capsular repair was reported in 5 of the 11 studies, with most repairing 100% of capsules. Follow‐up time ranged from 24 to 49.5 months.

The knot‐tying studies consisted of 425 patients (214 males, 211 females) with an average age of 36.7 years. Follow‐up time ranged from 25 to 60 months with an average of 40 months. The knotless studies consisted of 1084 patients (490 males, 594 females) with an average age of 36.9 years. Follow‐up time ranged from 12 to 37 months with an average follow‐up time of 28 months (Table 1).

mHHS was reported in 9 studies, HOS‐ADL in 5 studies, HOS‐SSS in 6 studies, VAS in 7 studies, International Hip Outcome Tool‐12 in 4 studies, and Nonarthritic Hip Score/UCLA Activity Score/Hip Disability and Osteoarthritic Outcome Score reported in 2 studies. Pre‐ and postoperative values for PROs for each study and suture subtype are reported in Table 1.

### PROs

Both knot‐tying and knotless groups showed significant improvement in mHHS scores after hip arthroscopy (Table 2). Mean differences and 95% confidence interval for each study are shown in Figure 2. The combined mHHS improvement in the knotless group was 24.05 ([95% confidence interval: 23.65‐24.44]; *I* ^2^: 3.1%; Figure 2). The combined mHHS improvement in the knot‐tying group was 18.69 (95% confidence interval: 15.67‐21.71; *I* ^2^: 94.5%; Figure 2). Similar analysis of pre‐ and postoperative differences in HOS‐ADL, HOS‐SSS, VAS, and International Hip Outcome Tool‐12 is shown in Figure S2.

**TABLE 2:** Follow‐Up Time, Number of Patients, Preoperative Patient‐Reported Outcome Measures, and Postoperative Patient Reported Outcomes Measures for Included Studies

**Author (Year)**	**Suture Anchor Type**	**Follow‐Up Time (Months)**	**Number of Patients**	**Preop mHHS (Mean [SD])**	**Postop mHHS (Mean [SD])**	**Preop HOS‐ADL (Mean [SD])**	**Postop HOS‐ADL (Mean [SD])**	**Preop HOS‐SSS (Mean [SD])**	**Postop HOS‐SSS (Mean [SD])**	**Preop VAS (Mean [SD])**	**Postop VAS (Mean [SD])**	**Preop IHOT12 (Mean [SD])**	**Postop IHOT12 (Mean [SD])**	**Preop NAHS (Mean [SD])**	**Postop NAHS (Mean [SD])**	**Preop UCLA (Mean [SD])**	**Postop UCLA (Mean [SD])**	**Preop HOOS (Mean [SD])**	**Postop HOOS (Mean [SD])**
Lim (2020)^18^	Knot‐tying	49.5	22	67.8 [15.7]	86.8 [5.5]	NR	NR	NR	NR	5.1 [2.1]	1.4 [1.4]	NR	NR	NR	NR	NR	NR	NR	NR
Vassalo (2018)^24^	Knot‐tying	43	80	80.4 [7.2]	96.6 [4.5]	NR	NR	NR	NR	NR	NR	NR	NR	NR	NR	NR	NR	NR	NR
Su (2024)^23^	Knot‐tying	31.2	76	67.7 [4.9]	86.3 [3.9]	64.2 [3.4]	83.9 [2.7]	42 [2.9]	72.6 [2.9]	6.2 [0.8]	2 [0.8]	NR	NR	NR	NR	NR	NR	NR	NR
Scanaliato (2022)^22^	Knot‐tying	60.12	76	66.1 [16.9]	81.23 [15.4]	NR	NR	NR	NR	NR	NR	39.8 [15.8]	73.44 [16.4]	NR	NR	NR	NR	NR	NR
Rhee (2016)^21^	Knot‐tying	32.3	19	66.6 [13.4]	78.5 [13.5]	50.9 [13.7]	63.1 [7.8]	66.9 [17.1]	75.2 [21.3]	5.9 [2.5]	2.3 [1.8]	NR	NR	NR	NR	3.2 [2.2]	6.0 [1.6]	44.1 [12.5]	57.4 [14.8]
Celiksoz (2024)^15^	Knot‐tying	25	157	63 [3.84]	86 [2.04]	39 [2]	86 [2]	39 [2]	86 [1]	5 [0.74]	2 [0.50]	35 [4.88]	80 [1.69]	NR	NR	NR	NR	NR	NR
Economopoulos (2020)^17^	Knotless	24	131	53.1 [12.85]	80.5 [18.03]	55 [17.32]	81.61 [10.01]	42.47 [23.14]	70.49 [12.43]	NR	NR	NR	NR	NR	NR	NR	NR	NR	NR
Christoforetti (2021)^16^	Knotless	12	162	NR	NR	63.9 [14.9]	88.7 [11.5]	45 [19.3]	78.8 [19.4]	5.76 [2.05]	1.94 [1.92]	34.5 [16.1]	75.1 [22.3]	NR	NR	NR	NR	NR	NR
Maldonado (2020)^19^	Knotless	30.59	309	62.6 [15.7]	86.9 [16.2]	NR	NR	39.8 [22]	74.2 [27.3]	5 [2.3]	2 [2.4]	36.7 [20.3]	77.6 [24.5]	63.1 [16.7]	86.1 [16.7]	NR	NR	NR	NR
Maldonado (2021)^20^	Knotless	37.6	106	61.43 [15.7]	85.59 [17.5]	NR	NR	NR	NR	4.7 [2.29]	1.82 [2.04]	NR	NR	62.04 [17.3]	86.19 [15.1]	NR	NR	NR	NR
Rhee (2016)^21^	Knotless	31.8	18	66.0 [19.7]	84.8 [9.2]	51.3 [17.7]	59.9 [12.4]	58.0 [19.9]	70.0 [27.0]	6.4 [1.5]	2.9 [2.2]	NR	NR	NR	NR	3.9 [1.5]	7.0 [2.4]	41.8 [15.1]	55.1 [15.2]
Celiksoz (2024)^15^	Knotless	34	256	62 [1.28]	86 [3.06]	39 [3]	86 [2]	39 [3]	87 [2]	6 [0.64]	1 [0.32]	35 [5.1]	79 [2.55]	NR	NR	NR	NR	NR	NR

HOS‐ADL, Hip Outcome Score‐Activities of Daily Living; HOS‐SSS, Hip Outcome Score‐Sport Specific Subscale; HOOS, Hip Disability and Osteoarthritic Outcome Score; IHOT12, International Hip Outcome Tool‐12; mHHS, modified Harris Hip Score; NAHS, Nonarthritic Hip Score; NR, not reported; SD, standard deviation; UCLA, UCLA Activity Score; VAS, Visual Analog Scale.

**FIGURE 2:**
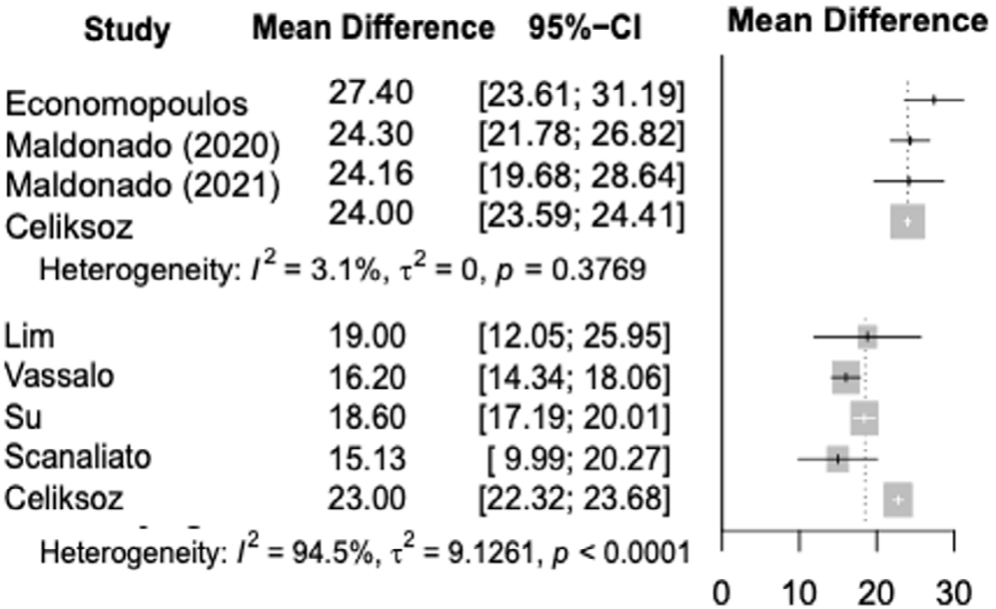
Forest plot showing the pre‐ and postoperative difference in mHHS score for each study grouped by knotless or knot‐tying suture anchor. (mHHS, modified Harris Hip Score.)

### Clinically Meaningful Outcome Achievement

Su et al.^23^ reported high and comparable rates of minimal clinically important difference (MCID) achievement between repair and selective debridement groups across multiple PROs, including HHS (85.5% vs 84.2%), HOS‐ADL (88.2% vs 89.5%), and HOS‐SSS (84.2% vs 86.8%). Patient acceptable symptom state (PASS) achievement for HHS was reached by 100% of repair patients and 82.9% of selective debridement patients, whereas PASS rates for HOS‐ADL and HOS‐SSS were lower and ranged from approximately 49% to 55%, with no statistically significant differences between groups.^23^ Celiksoz et al.^15^ showed similarly high rates of clinically meaningful improvement, with MCID achievement exceeding 99% and PASS exceeding 97% for HOS‐ADL, HOS‐SSS, mHHS, and iHOT‐12 in both knotless and knot‐tying anchor groups; for VAS pain, MCID achievement was 99.6% in the knotless group and 96.8% in the knot‐tying group, whereas PASS was achieved by 46.1% and 65.0%, respectively.^15^

Scanaliato et al.^22^ reported MCID achievement exceeding 95% for both iHOT‐12 and mHHS after labral repair and reconstruction, with PASS achievement ranging from approximately 73% to 79% and substantial clinical benefit achieved in 53% to 57% of patients for iHOT‐12 and 35% to 40% for mHHS, without significant differences between cohorts. Maldonado et al.^19^ similarly reported MCID and PASS achievement rates of 78.6% and 82.2% for mHHS, 60.8% and 69.9% for HOS‐SSS, and MCID achievement of 77.3% for iHOT‐12. In a subsequent propensity‐matched analysis, Maldonado et al.^20^ showed comparable MCID and PASS achievement between labral repair and reconstruction across mHHS, Nonarthritic Hip Score, and VAS pain, with MCID and PASS rates generally ranging from approximately 60% to 76% and no statistically significant between‐group differences.

### Revision, Arthroplasty Conversion, and Complications

Surgical revision rate and conversion to a THA were the 2 most common modes of failure referenced in the 10 included studies. Revision rate was reported in 3 out of 6 knot‐tying studies and ranged from 0% to 12.8%. Revision rate was reported in 4 out of 6 knotless groups and ranged from 3.3% to 6.8% (Table 3). Likewise, THA conversion was only reported in half of the knot‐tying and knotless groups, ranging from 0% to 4.7% in knot‐tying and 2.6% to 5.7% in knotless (Table 3). Celiksoz et al. directly compared complication profile between knotless and knot‐tying, showing a higher occurrence of synovitis (knot‐tying: 17/157; knotless: 5/256) and suture reaction (knot‐tying: 13/157; knotless: 5/256) in the knot‐tying group, but increased paresthesia (knotless: 11/256; knot‐tying: 4/157) and stiffness (knotless: 7/256; knot‐tying: 2/157) in the knotless group (Table 3). Only synovitis was deemed statistically significant (*P* = .01). Other studies reported rare occurrences of neuropraxias and iatrogenic cartilage injury (Table 3).

**TABLE 3:** Complication Profile, Revision Rate, and Rate of Conversion to Total Hip Arthroplasty Among Included Studies

**Author (Year)**	**Suture Anchor Type**	**Follow‐Up Time**	**Complications**	**Revision Rate (n (%))**	**Conversion to THA (n (%))**
Lim (2020)^18^	Knot‐tying	4 yr	Iatrogenic labral perforation and cartilage injury (n = 3)	NR	NR
Vassalo (2018)^24^	Knot‐tying	43 mo	NR	NR	4 (4.7%)
Su (2024)^23^	Knot‐tying	2 yr	Temporary neuropraxia of pudendal nerve (n = 2)	0 (0%)	0 (0%)
Scanaliato (2022)^22^	Knot‐tying	2 yr	NR	2 (2.94%)	1 (1.47%)
Rhee (2016)^21^	Knot‐tying	2 yr	Drill penetration into subchondral area (n = 1)	NR	NR
Celiksoz (2024)^15^	Knot‐tying	2 yr	Paresthesia (n = 4) Perineal skin injury (n = 3) Stiffness (n = 2) Suture Reaction (n = 13) Synovitis (n = 17)	5 (12.8%)	NR
Economopoulos (2020)^17^	Knotless	2 yr	NR	8 (5.3%)	4 (2.6%)
Christoforetti (2021)^16^	Knotless	1 yr	NR	NR	NR
Maldonado (2020)^19^	Knotless	2 yr	NR	21 (6.8%)	8 (2.6%)
Maldonado (2021)^20^	Knotless	2 yr	NR	2 (1.9%)	6 (5.7%)
Rhee (2016)^21^	Knotless	2 yr	Drill penetration into subchondral area (n = 2)	NR	NR
Celiksoz (2024)^15^	Knotless	2 yr	Paresthesia (n = 11) Perineal skin injury (n = 2) Stiffness (n = 7) Suture Reaction (n = 5) Synovitis (n = 5)	1 (3.3%)	NR

mo, months; NR, not reported; THA, total hip arthroplasty; yr, years.

## DISCUSSION

In the treatment of acetabular labrum tears, patients can expect a high likelihood of clinical improvement with a similar complication and THA conversion profile, regardless of the suture anchor type used. Ten studies were included in our analysis. Given the substantial heterogeneity among the studies, we did not perform a meta‐analysis, although our data strongly suggest greater improvement in mHHS scores in the knotless suture group compared with the knot‐tying group. There was no significant difference in improvement for other PROs. Complication profiles were favorable and similar between knotless and knot‐tying suture anchors. There was no significant difference in revision rate or conversion to THA. Notably, when clinical relevance was assessed using patient‐level thresholds, most of patients in both knotless and knot‐tying groups achieved MCID and PASS across commonly reported PROs, suggesting that the observed improvements translate into meaningful symptomatic benefit for most patients rather than solely statistical change. Although our analysis of mHHS scores showed a difference between knotless and knot‐tying groups, correlating group‐level differences to clinical relevance remains challenging. Although multiple included studies reported high proportions of patients achieving MCID and PASS, these thresholds varied across outcome measures and study methodologies, limiting direct comparison and precluding pooled analysis of clinically meaningful outcome achievement. The MCID for hip arthroscopy has varied widely in the literature. A recent systematic review found that MCID for the mHHS ranges from 2.4 to 16.8 for distribution‐based methods and 7.2 to 20.9 for anchor‐based methods, calling into question the actual clinical benefit seen with knotless anchors in this study.^25^

Currently, the use of knotless versus knot‐tying suture anchors for hip labral repair in the setting of FAI often falls into surgeon preference without strong data supporting the use of one type over the other. A recent systematic review from Johns et al. also compared PROs in knotless versus knot‐tying suture anchors in hip labral repairs and did not find significant differences between the 2 groups.^26^ However, Johns et al. included studies in which patients also underwent procedures such as labral reconstruction that could potentially impact PROs.

The studies by Rhee et al. and Celiksoz et al. directly compared knotless and knot‐tying suture anchors in a randomized control trial and prospective cohort study, respectively.^15^, ^21^ Although Rhee et al. did not find any significant differences between the 2 groups at 2‐year follow‐up, Celiksoz noted improved HOS‐SSS and VAS pain scores in the knotless group, as well as fewer patients with perioperative synovitis. Although our data did not reflect similar improvements in HOS‐SSS and VAS pain scores, the higher improvement in mHHS scores for knotless groups does suggest a potential advantage of knotless suture anchors over traditional knot‐tying suture anchors.

Proponents of knotless suture anchors have proposed several theoretical advantages over traditional knot‐tying, including reduced surgical time and complexity, biomechanical advantages, and lower risk of knot‐related complications.^10^, ^11^, ^27^ Benca et al. showed that knotless anchors have similar biomechanical properties and have a lower insertion time compared with knotted anchors,^10^ and Rhee et al. also noted a shorter traction time in their knotless group.^21^ Knotless anchors have also been shown to provide minimal displacement at physiological loads, which can be beneficial for maintaining the stability of the repair during the early phases of healing and better outcomes.^11^ Furthermore, knotless anchors could allow for more consistent tension and reproducibility by eliminating inherent variability with manual knot‐tying, although this is surgeon‐dependent as some surgeons feel that knot tension can be more reliably established with a knot‐tying anchor. Knotless anchors also avoid the potential issues associated with knot stacks, such as knot abrasion, which can lead to soft‐tissue or cartilage damage.^27^

### Limitations

There are multiple important limitations within our study. First, there were over twice as many subjects with knotless anchors (1084) than knotted (425). This can skew the results. Our systematic review also only included studies that explicitly indicated specific suture anchor used, excluding several large cohort studies that met all other inclusion criteria. Furthermore, we used strict inclusion criteria and evaluated each study using the population, intervention, comparison, and outcome framework. Accordingly, we only examined acetabular labral repairs in the setting of FAI, making our results less applicable to other areas of hip arthroscopy (i.e., labral repairs in the setting of hip dysplasia or labrum reconstruction).

We also recognize the high clinical and statistical heterogeneity among our study. This is evident from the different clinical questions asked in each study included in our review, including studies on capsular management techniques, hypertrophic versus morphologically normal labra, and patients older than 40 years Finally, as shown in our risk assessment, many of our studies had potential risks of bias, especially confounding bias (6/8 studies) and selection bias (7/8 studies). We understand that this can influence the results and clinical applicability of this study.

## CONCLUSIONS

Knotless and knot‐tying suture anchors are both safe and effective options for hip labral repairs with anticipated improvement in PROs regardless of the anchor type used. Across studies reporting patient‐level thresholds, a high proportion of patients achieved MCID and PASS, supporting the clinical relevance of postoperative improvement in both constructs. There were no significant differences in revision rates or conversion to THA, and complications are low in both groups.

## SUPPORTING INFORMATION

Additional supporting information can be found online in the Supporting Information section.

## DISCLOSURES

The authors (D.H., L.N., S.R.J., P.E., U.S., V.K.T.) declare that they have no known competing financial interests or personal relationships that could have appeared to influence the work reported in this article.

## Supporting information

Supplementary Material
