# Supplementary material for: Both Knotless and Traditional Knot‐Tying Suture Anchors Result in Favorable Clinical Outcomes in the Treatment of Acetabular Labral Tears: A Systematic Review
Source: Arthrosc Sports Med Rehabil. 2026 Sep 28;8(5):e70092. doi: 10.1002/ars2.70092 (PMC13620422; doi:10.1002/ars2.70092)
Supplement: Supplementary file 1 [file ARS2-8-e70092-s001.pdf]

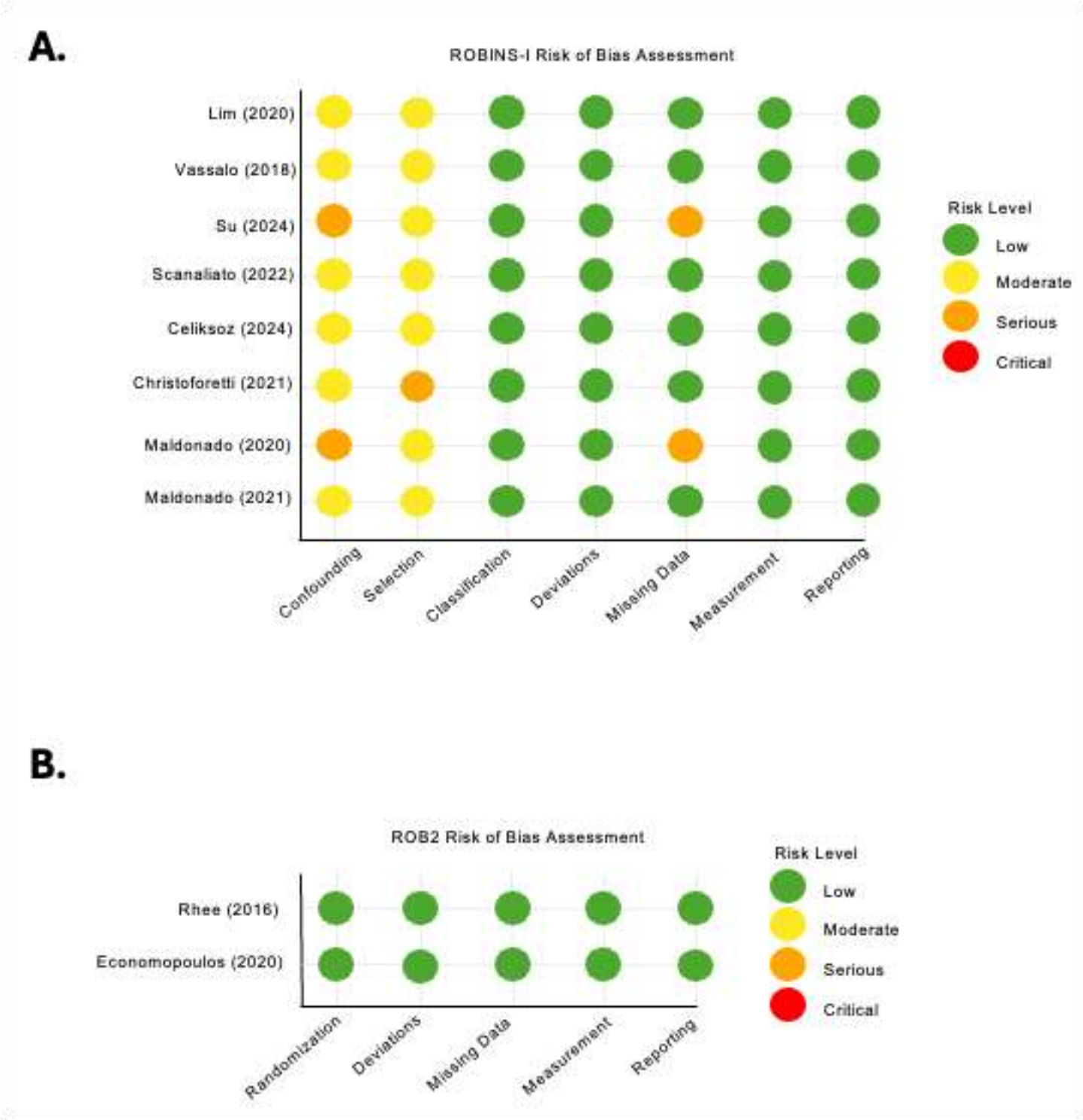

Supplemental Figure 1. Risk of bias assessment for included studies, including A) ROBINS-I tool for nonrandomized studies and B) RoB 2.0 tool for randomized controlled trials.

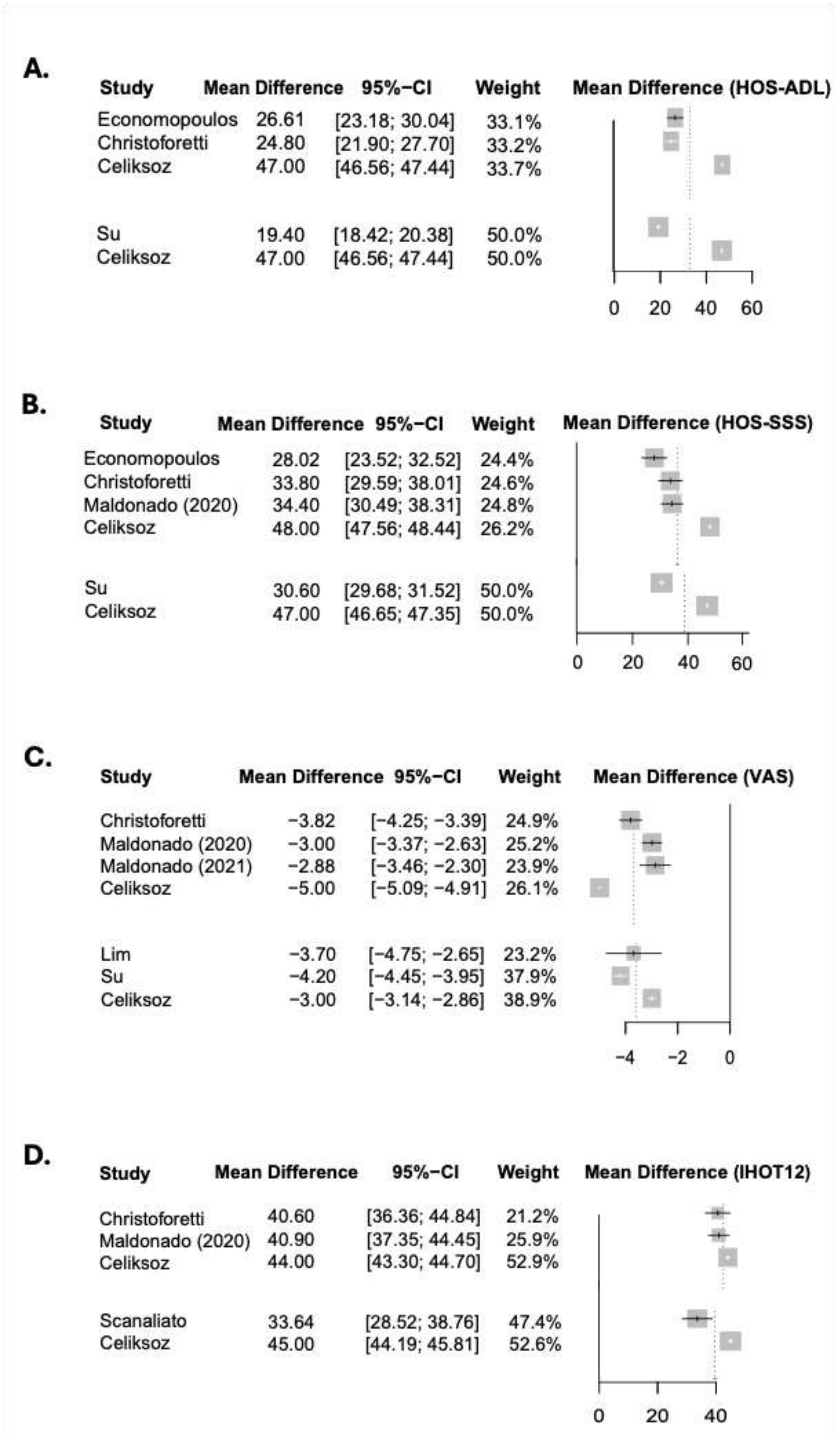

Supplemental Figure 2. Forest plot showing the pre-op and post-op difference in various patient-reported outcome scores for each study as well as pooled pre-op and post-op changes for knotless and knot-tying groups, including A) HOS-ADL, B) HOS-SSS, C) VAS, and D) iHOT12.
